# miR-126 Mimic Counteracts the Increased Secretion of VEGF-A Induced by High Glucose in ARPE-19 Cells

**DOI:** 10.1155/2021/6649222

**Published:** 2021-02-24

**Authors:** Roberta Sanguineti, Alessandra Puddu, Massimo Nicolò, Carlo Enrico Traverso, Renzo Cordera, Giorgio L. Viviani, Davide Maggi

**Affiliations:** ^1^Department of Internal Medicine and Medical Specialties, Viale Benedetto XV XV, Genova, Italy; ^2^Department of Neuroscience, Ophthalmology and Genetics, Viale Benedetto, Genova, Italy; ^3^Fondazione per la Macula Onlus–Genova, Piazza della Vittoria, Genova, Italy

## Abstract

Vascular endothelial growth factor-A (VEGF-A) has a pathologic role in microvascular diabetic complication, such as diabetic retinopathy (DR). miR-126 plays an important role in vascular development and angiogenesis by regulating the expression of VEGF-A. Since levels of miR-126 have been found downregulated in diabetes, this study is aimed at investigating whether hyperglycemia affects expression of miR-126 in a retinal pigment epithelium cell line. ARPE-19 cells were transfected with miR-126 inhibitor or with miR-126 mimic and the respective scramble negative control. After 24 hours, medium was replaced and cells were cultured for 24 hours in normal (CTR) or diabetic condition (HG). Then, we analyzed mRNA levels of miR-126, VEGF-A, PI3KR2, and SPRED1. We also evaluated protein amount of HIF-1*α*, PI3KR2, and SPRED1 and VEGF-A secretion. The results showed that exposure of ARPE-19 cells to HG significantly decreased miR-126 levels; mRNA levels of VEGF-A and PI3KR2 were inversely correlated with those of miR-126. Overexpression of miR-126 under HG restored HIF-1*α* expression and VEGF-A secretion to the level of CTR cells. These results indicate that reduced levels of miR-126 may contribute to DR progression by increasing expression of VEGF-A in RPE cells. In addition, we provide evidence that upregulation of miR-126 in RPE cells counteracts the rise of VEGF-A secretion induced by hyperglycemia. In conclusion, our data support a role of miR-126 mimic-approach in counteracting proangiogenic effects of hyperglycemia.

## 1. Introduction

Diabetic retinopathy (DR) is one of the most important microvascular complications of diabetes and the primary cause of visual loss in working age adults [1–3]. Prolonged hyperglycemia is a significant risk factor in the DR progression and could cause ocular neovascularization with aberrant formation of immature blood vessels [4, 5]. Indeed, it leads to progressive alterations of the retinal microvasculature, which start with pericyte dropout, pass through vasoregression and increased vasopermeability, and lead to pathological neovascularization in response to hypoxia [6].

Vascular endothelial growth factor-A (VEGF-A), a key mediator of blood vessel formation, plays an important role in the homeostasis of the retinal and choroidal vasculature, by mediating both angiogenesis and inflammation [7]. Hyperglycemia induces aberrant levels of VEGF-A in the retina, which have been related to structural and functional changes that lead to DR [8, 9]. Indeed, VEGF-A activates quiescent endothelial cells, promotes cell proliferation and migration with the subsequent formation of new blood vessels, and increases vascular permeability [10].

The retinal pigment epithelium (RPE) is a monolayer of highly specialized cells located between the choroid and photoreceptors, which form the outer blood-retinal barrier (BRB) [11]. RPE cells play an important role in retinal homeostasis, by affecting the function and maintenance of both the photoreceptors and capillary endothelium. Consequently, RPE barrier dysfunction and altered secretion of growth factors and cytokines by RPE cells contribute to diabetic retinopathy worsening. In particular, RPE cells are one of the main source of VEGF-A in the retina. During homeostasis, VEGF-A is secreted in low concentrations to the basal side of the RPE, where it contributes in maintaining endothelial cell survival and choriocapillaris fenestrations. Under diabetic conditions, VEGF-A is overproduced and released also toward the apical side [12], thus increasing the permeability of the choroidal vessels in diabetic eyes and compromising the maintenance of the structural integrity of the retina.

It is well known that transcription of VEGF-A is regulated by hypoxia-inducible factor 1-alpha (HIF-1*α*), a transcription factor that is the master regulator of cellular response to hypoxia and hyperglycemia [9, 13]. Briefly, HIF-1 is a heterodimeric transcription factor consisting of a constitutively expressed *β*-subunit and an oxygen-regulated *α*-subunit [14]. Under normoxic conditions, HIF-1*α* is degraded by proteasomes; on the contrary, in hypoxic conditions and during hyperglycemia, HIF-1*α* is stabilized and able to interact with its coactivators and the *β*-subunit to increase expression of genes involved in energy metabolism and angiogenesis, including VEGF-A.

Expression of VEGF-A may be also regulated by microRNAs, small noncoding RNAs that regulate gene expression at posttranscriptional level [15]. In particular, miR-126, an endothelial specific microRNA, has been reported to have a central role in neovascularization process by regulating VEGF-A signaling [16]. Indeed, miR-126 targets to a binding site in 3'UTR of VEGF-A mRNA; therefore, reduced levels of miR-126 lead to increased expression of VEGF-A and may promote vascular permeability and pathological vascularization; on the contrary, miR-126 overexpression decreases the levels of VEGF-A [17]. In addition, miR-126 may affect the VEGF/PI3K/AKT signaling pathway targeting the expression of Sprouty-related protein (SPRED1) and phosphoinositol-3 kinase regulatory subunit 2 (PI3KR2), which are two negative regulators of VEGF-A expression [16, 18, 19].

Since the RPE cell dysfunction is involved in the early stages of the DR damage and miR-126 represents a promising target for novel antiangiogenic therapies, this study is aimed at investigating whether hyperglycemia affects expression of miR-126 and characterizing the molecular mechanisms through which miR-126 regulates VEGF-A expression in RPE cells.

## 2. Materials and Methods

### 2.1. Cell Culture and Experimental Conditions

The human cell line ARPE-19 (American Type Culture Collection, Manassas, VA, USA) from passages 22 to 28 were grown in DMEM/F12 1 : 1 medium (Euroclone, Milan, Italy) supplemented with 10% fetal bovine serum and 2 mmol/L glutamine (Euroclone, Milan, Italy) at 37°C in 5% CO_2_. The cell medium was replaced every 2 days. Cells were grown to confluence, removed with trypsin-EDTA (Euroclone, Milan, Italy), and then seeded in multiwell plates for all experiments. Before each experiment, confluent cells were washed with phosphate-buffered saline (PBS) (Euroclone, Milan, Italy) and cultured in control medium (DMEM low glucose/F12, CTR).

### 2.2. miR-126 Mimics/Inhibitor Transfection

ARPE-19 cells were plated into 12-well plates with 8 × 104 cells/well and cultured in normal glucose condition. Once the cells were 70% confluent, has-miR-126-3p miRCURY LNA Power inhibitor (miR-126 inhib), has-miR-126-3p miRCURY LNA Mimic (miR-126 mimic), and the respective scramble negative controls (all from Exiqon-Qiagen, Milan, Italy) were transfected into ARPE-19 cells using jetPRIME transfection reagent (Polyplus-transfection, New York, USA). After 24 hours of the transfection, medium was changed and replaced with fresh CTR or high glucose (HG, 25 mM glucose) medium for 24 hours.

### 2.3. Quantitative RT-PCR

RNA was extracted from ARPE-19 cells cultured under different conditions using the Quick-RNA Mini prep Kit (Zymo Research, Irvine, CA) according to the manufacturer's instructions. The amount and quality of RNA were determined spectrophotometrically. RNA samples featuring an A260/A280 value of at least 2.0 were generally used for further analysis. To analyze miRNA-expression level, 5 ng/*μ*L of total RNA was reversed-transcribed using miRCURY LNA RT kit (Exiqon-Qiagen, Milan, Italy). The obtained cDNA was then diluted for further quantitative real-time polymerase chain reaction (qRT-PCR). miRNA levels were measured using miRCURY LNA miRNA PCR Assay (Exiqon-Qiagen, Milan, Italy) with specific primers: has-miR-126-3p and U6 snRNA(has, mmu) used as a normalization control for miRNA expression. All measurements were performed in triplicate on an ABI PRISM 7900 HT Fast Real-Time PCR System (Applied Biosystems Monza, Italy). Comparisons in gene expression were done using the 2^−*ΔΔ*Ct^ method [20].

For mRNA analysis, one microgram of RNA was reverse-transcripted to cDNA using Wonder RT-cDNA Synthesis kit (Euroclone, Milan, Italy). The expression levels of the target gene VEGF-A (Applied Biosystems assay ID: Hs00900055_m1) were measured by qRT-PCR amplification, performed using Luna Universal Probe qPCR Master Mix (New England Biolabs, NEB, Massachusetts, USA) in an ABI PRISM 7900 HT Fast Real-Time PCR System (Applied Biosystems Monza, Italy). All measurements were performed in triplicate with the following qRT-PCR run protocol: initial denaturation program (95°C 1 min), denaturation (95°C 15 sec), and extension program (60°C 30 sec) repeated 43 times (95°C 15 s and 60°C 1 min). Gene expression was normalized using the housekeeping as control gene (*β*-actin, Applied Biosystems assay ID: Hs01060665_g1). Comparisons in gene expression were done using the 2^−*ΔΔ*Ct^ method [20].

### 2.4. Secretion of VEGF-A

ARPE-19 cells were treated with miRNA mimic or miRNA inhibitor for 48 hours. At the end of incubation, fresh CTR or HG medium was added for 24 h. To quantify VEGF-A secretion, the conditioned media were collected and stored at −80°C until the assay was performed. Cells were then washed with PBS and lysed in radioimmunoprecipitation assay (RIPA) buffer, and protein content was determined with the BCA Protein Assay Kit (Pierce, Rockford, MD) according to the manufacturer's instructions. Secretion of VEGF-A was assessed with enzyme-linked immunosorbent assay (ELISA; Raybiotech, Norcross, GA, USA), and concentrations were calculated from the standard curve and normalized to the total protein concentration of the respective lysate.

### 2.5. Immunoblot

At the end of the experiments, ARPE-19 cells were lysed in RIPA buffer supplemented with protease and phosphatase inhibitors, and protein concentrations were determined using the BCA Protein Assay Kit. Thirty micrograms of total cell lysate were separated on a SDS–PAGE and transferred onto nitrocellulose. Filters were blocked in 5% nonfat dried milk and incubated overnight at 4°C with primary specific antibodies (HIF-1*α*, PI3KR2, and SPRED1 from Cell Signaling Technology, Beverly, MA, USA). Secondary specific horseradish peroxidase-linked antibodies were added for 1 h at room temperature. Bound antibodies were detected using the enhanced chemiluminescence lighting system (ECL Plus), according to the manufacturer's instructions. Each membrane was stripped (Restore PLUS Western Blot Stripping Buffer, Pierce Biotechnology, Rockford, IL, USA) and probed for *β*-actin (Cell Signaling Technology, Beverly, MA, USA) to verify equal protein loading. Bands of interest were quantified by densitometry using the Alliance software. Results were expressed as percentages of CTR (defined as 100%).

### 2.6. Statistical Analysis

All statistical analyses were performed using the GraphPad Prism 4.0 software (GraphPad Software, San Diego, CA, USA). Data were expressed as the mean ± SEM and then analyzed using one-way ANOVA followed by Dunnett's multiple comparison test and *t*-test. A *p* value of <0.05 was considered statistically significant. The results are representative of at least 3 experiments.

## 3. Results

### 3.1. Hyperglycemia Decreased miR-126 Expression

First, we performed RT-qPCR to confirm the presence of miR-126 in ARPE-19 cells and the efficiency of transfection with miR-126 inhibitor and mimic.

**Figure 1:**
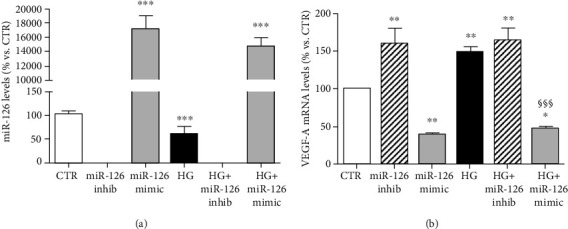
(a) Levels of miR-126 in ARPE-19 cells cultured for 24 hours in standard medium (CTR), silencing (inhib) and upregulating (mimic) miR-126 in control condition and in presence of high glucose (HG). Gene expression was normalized vs. U6 housekeeping gene. (b) mRNA levels of VEGF-A in ARPE-19 cells cultured for 24 hours in standard medium (CTR), silencing (inhib) and upregulating (mimic) miR-126 in control condition and in presence of high glucose (HG). VEGF-A gene expression was normalized vs. *β*-actin as housekeeping gene. Data are presented as the mean ± SEM of three experiments (*n* = 3). ^∗^*p* < 0.05, ^∗∗^*p* < 0.01, and ^∗∗∗^*p* < 0.001 vs. CTR; ^$$$^*p* < 0.001 HG+mimic vs. HG.

As expected, miR-126 was expressed by ARPE-19 cells (Figure 1(a)). Hyperglycemia significantly reduced miR-126 levels compared to control conditions (Figure 1(a)). Addition of miR-126 inhibitor completely abrogated the detection of miR-126 in the samples. On the contrary, transfection of miR-126 mimic significantly increased miR-126 levels.

### 3.2. mRNA Levels of VEGF-A Are Inversely Correlated to Those of miR-126 under Standard and Diabetic Conditions

Addition of miR-126 inhibitor or mimic resulted, respectively, in a significant upregulation or downregulation of VEGF-A gene expression under control conditions.

In presence of high glucose, the VEGF-A gene expression was significantly increased (Figure 1(b)). Treatment with miR-126 inhibitor did not further increase the effect of hyperglycemia on VEGF-A expression (Figure 1(b)). Transfection of miR-126 mimic prevented the rise of VEGF-A expression levels induced by HG.

### 3.3. HIF-1*α* Protein Expression Is Not Affected by Levels of miR-126

HIF-1*α* is the main regulator of VEGF-A. To determine its contribution on VEGF-A expression when miR-126 is down- or upregulated, we evaluated HIF-1*α* protein expression.

**Figure 2:**
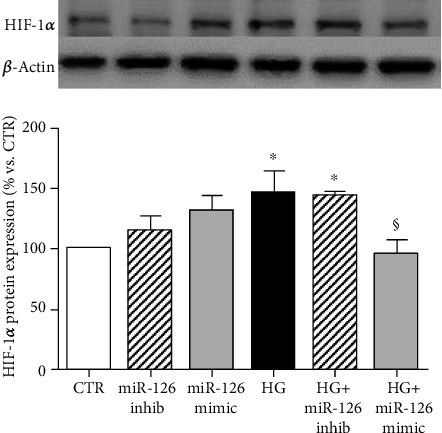
Western blot analysis of HIF-1*α* protein levels in ARPE-19 cells cultured for 24 hours in standard medium (CTR), silencing (inhib) and upregulating (mimic) miR-126 in control condition and in presence of high glucose (HG). Representative Western blot analysis and quantification of densitometries of Western blot band. Data were expressed as mean ± SEM of fold induction relative to *β*-actin of three independent experiments (*n* = 3). ^∗^*p* < 0.05 vs. CTR; ^$^*p* < 0.05 HG+mimic vs. HG.

Treatment with HG significantly increased HIF-1*α* protein level. Under control condition, no differences in HIF-1*α* protein expression were observed in ARPE-19 cells transfected either with miR-126 inhibitor or miR-126 mimic (Figure 2). Transfection with miR-126 inhibitor did not affect HG-induced upregulation of HIF-1*α*. On the contrary, transfection with miR-126 mimic caused a significant downregulation of HIF-1*α* expression compared to HG cultures (Figure 2).

### 3.4. miR-126 Targeted SPRED1 and PI3KR2 in ARPE-19 Cells

In endothelial cells, miR-126 may regulate VEGF-A levels by directly repressing SPRED1 and PI3KR2, two negative regulators of the VEGF pathway [16, 18, 19]. To investigate whether SPRED1 and PI3KR2 are targets of miR-126 in ARPE-19 cells, we performed RT-qPCR and Western blot analysis in ARPE-19 cells transfected with miR-126 inhibitor or miR-126 mimic at normal and high-glucose conditions.

**Figure 3:**
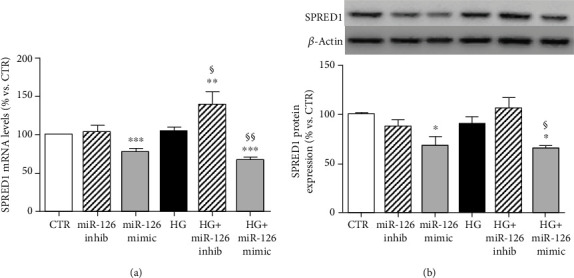
Expression of SPRED1 in ARPE-19 cells cultured for 24 hours in standard medium (CTR), silencing (inhib) and upregulating (mimic) miR-126 in control condition and in presence of high glucose (HG). (a) SPRED1 gene expression normalized vs. *β*-actin as housekeeping gene. (b) Representative Western blot analysis of SPRED1 protein expression with quantification of densitometries of relative bands. Data were expressed as mean ± SEM of fold induction relative to *β*-actin of three independent experiments (*n* = 3). ^∗^*p* < 0.05, ^∗∗^*p* < 0.01, and ^∗∗∗^*p* < 0.001 vs. CTR; ^§^*p* < 0.05 and ^§§^*p* < 0.01 HG+mimic vs. HG.

SPRED1 gene and protein expression were not affected by hyperglycemia (Figures 3(a) and 3(b)). The inhibition of miR-126 did not alter SPRED1 gene and protein expression under control conditions, but induced a significant increase of SPRED1 mRNA level under hyperglycemic conditions. Transfection with miR-126 mimic significantly downregulated SPRED1 gene and protein expression.

**Figure 4:**
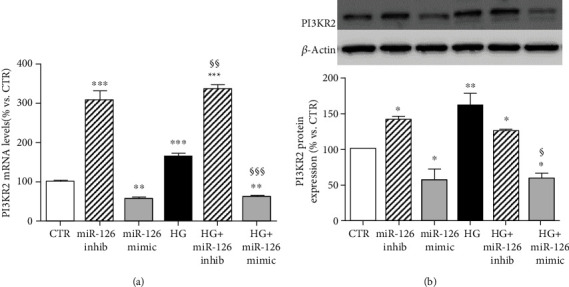
Expression of PIK3R2 in ARPE-19 cells cultured for 24 hours in standard medium (CTR), silencing (INHIB) and upregulating (MIMIC) miR-126 in control condition and in presence of high glucose (HG). (a) PIK3R2 gene expression normalized vs. *β*-actin as housekeeping gene. Representative Western blot analysis of (b) PIK3R2 protein expression with quantification of densitometries of relative bands. Data were expressed as mean ± SEM of fold induction relative to *β*-actin of three independent experiments (*n* = 3). ^∗^*p* < 0.05, ^∗∗^*p* < 0.01, and ^∗∗∗^*p* < 0.001 vs. CTR; ^§^*p* < 0.05, ^§§^*p* < 0.01, and ^§§§^*p* < 0.001 HG+mimic vs. HG.

High glucose significantly enhanced PI3KR2 gene and protein expression compared to control (Figures 4(a) and 4(b)). When miR-126 was inhibited, PI3KR2 gene and protein expression were upregulated in comparison to control cultures. On the contrary, overexpression of miR-126 significantly reduced the mRNA and protein levels of PI3KR2 and prevented the rise induced by HG.

### 3.5. Overexpression of miR-126 Restores VEGF-A Secretion under HG to the Level of CTR Cells

**Figure 5:**
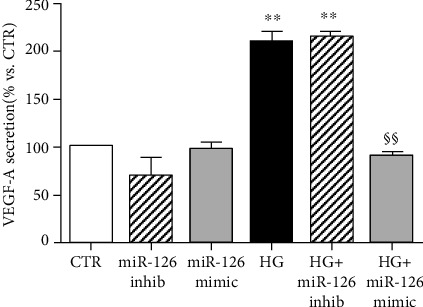
VEGF-A secretion measured by ELISA analysis. Data are presented as the mean ± SEM of three experiments (*n* = 3). ^∗∗^*p* < 0.01 vs. CTR; ^$§^*p* < 0.01 HG+mimic vs. HG.

Finally, we investigated whether the potential of therapeutic strategy based on miR-126 enrichment may counteract the increased secretion of VEGF-A induced by hyperglycemia. VEGF-A release was not affected neither by inhibition nor by overexpression of miR-126. Treatment with HG alone or in combination with miR-126 inhibitor increased VEGF-A secretion compared with control culture (Figure 5). miR-126 mimic transfection prevented the rise of VEGF-A secretion induced by HG, maintaining the release to the levels observed in control culture.

## 4. Discussion

Chronic hyperglycemia is a major long-term determinant of vascular changes in DR. Retinal neovascularization is primarily due to uncontrolled VEGF-A expression and secretion by the RPE [8, 11]. It is well known that the expression of VEGF-A is regulated by the activity of HIF-1*α*, which is stabilized under hypoxia and diabetes [9, 13]. VEGF-A expression is also controlled by miR-126 [16]. Therefore, it is important to determine whether glucose may affect levels of miR-126 in RPE cells.

The mechanisms through which miR-126 regulates angiogenesis have been deeply investigated in endothelial cells and are mainly related to modulation of VEGF-A expression [16]. Recently, Zhou et al. showed that miR-126 is involved in the regulation of VEGF-A expression and also in RPE cells suggesting that VEGF-A is a miR-126-3p target [21]. Consistent with this report, we found that levels of miR-126 are inversely correlated with VEGF-A expression in ARPE-19 cells. To further explore whether altered expression of VEGF-A is due to a direct action of miR-126 on its mRNA transcript, we investigated the expression of HIF-1*α*. Under physiologic condition, treatment with miR-126 inhibitor or mimic, respectively, increased or reduced mRNA levels of VEGF-A without affecting protein expression of HIF-1*α*. Usually, the expression of HIF-1*α* correlates with mRNA levels of VEGF-A. However, we found that in normal condition, the amount of HIF-1*α* does not affect mRNA levels of VEGF-A. This suggests that reduction of VEGF-A mRNA in ARPE-19 cells is due to mRNA degradation induced by interaction with miR-126. On the contrary, the rise of VEGF-A expression may be related to lack of induction of degradation. Taken together, our results confirm that VEGF-A is a target of miR-126 in ARPE-19 cells.

The presence of a relationship between miR-126 levels and diabetes has been suggested by several studies. Plasma levels of miR-126 are lower in patients with DM in comparison with healthy subjects. [22]. Furthermore, levels of miR126 have been found downregulated in the retinal tissue of streptozotocin-induced diabetic rat [23]. Consistent with this evidence, we found that hyperglycemia decreased levels of miR-126, demonstrating that glucose may regulate miR-126 expression in RPE cells. Moreover, under hyperglycemia, reduced levels of miR-126 are coupled to rise of VEGF-A mRNA levels and increased the expression of HIF-1 *α*. Taken together, these findings suggest that both reduced degradation of VEGF-A mRNA and increased transcription of its gene contribute to upregulate VEGF-A mRNA levels. Use of miR-126 inhibitor did not further increase the levels of VEGF-A, reached when cells are cultured under diabetic condition, suggesting that hyperglycemia induced the maximum levels of VEGF-A expression. On the contrary, miR-126 mimic prevented the upregulation of HIF-1*α* expression and counteracted the increased levels of VEGF-A induced by hyperglycemia, suggesting that HIF-1*α* may be an indirect target of miR-126 in ARPE-19 cells.

It has been shown that miR-126 regulates VEGF-A expression not only by directly targeting VEGF-A, but also by regulating the levels of SPRED1 and PI3KR2 [16, 18, 19]. Yang et al. demonstrated that miR-126 regulated VEGF-A and PI3KR2 in retinal vascular endothelial cells under diabetic condition, and that miR-126 overexpression blocked the cell migration and sprouting induced by high glucose by inhibiting VEGF-A expression [19]. In addition, Wang et al. showed that transfection with miR-126 mimic prevented the increased mRNA and protein levels of VEGF-A and SPRED1 under diabetic condition [24]. Consistent with these findings, we observed an inverse correlation between levels of miR-126 and those of SPRED1 when cells are transfected with miR-126 mimic, both under control and high-glucose conditions, confirming that SPRED1 is a target of miR-126 in ARPE-19 cells. Since depletion of miR-126 did not result in upregulation of SPRED1 protein expression, it is likely that this is the maximum amount of SPRED1 produced by ARPE-19 cells. Furthermore, our results showed that miR-126 levels are inversely correlated with expression of PI3KR2 in all experimental conditions, demonstrating that in ARPE-19 cells, miR-126 directly targets PI3KR2. Taken together, these data suggest that SPRED1 and PI3KR2 are regulated by miR-126 and also in ARPE-19 cells. However, different from findings in other cell types [18, 25], the downregulation of SPRED1 and PI3KR2 expression did not result in increased levels of VEGF-A, suggesting that these two factors are not involved in the regulation of VEGF-A expression in ARPE-19 cells, and that miR-126 function is highly tissue specific. Reduced levels of miR-126 due to hyperglycemia play a crucial role in the pathogenesis and progression of DR [26]. VEGF-A is the main target of most pharmacological interventions to prevent the DR progression. However, the increased expression of VEGF-A in RPE cells could be also countered through an innovative therapeutic strategy represented by miR-126 enrichment. In this regard, several studies are underway to define a role for miR-126 overexpression as a future gene therapy to counteract the abnormal VEGF-A expression levels, thus offering new prospective in clinical application [26, 27]. Here, we found that overexpression of miR-126 counteracts the rise in VEGF-A secretion induced by hyperglycemia. To our knowledge, this is the first evidence that miR-126 overexpression may reduce secretion of VEGF-A in diabetic condition in RPE cells. Of importance, miR-126 mimic did not affect VEGF-A secretion under control condition, suggesting that this approach did not alter homeostasis in normoglycemic environment. Furthermore, this result suggests that downregulation of VEGF-A secretion under diabetic condition may be related to the decrease production of VEGF-A due to a reduced mRNA expression rather than to a defect in its release. Secretion of VEGF-A has not changed even in the presence of increased expression of VEGF-A due to the effect of miR-126 inhibitor, confirming that the secretory machine of ARPE-19 cells is not affected neither by the levels of miR-126 nor by those of VEGF-A.

## 5. Conclusions

In conclusion, we demonstrated that miR-126 overexpression may be useful in reducing VEGF-A secretion from RPE cells under diabetic condition. This, in turns, may prevent progressive alterations of the retinal microvasculature by reducing both vascular permeability and activation of endothelial cells. Considering that RPE cell dysfunction is one of the early changes in the onset of diabetic retinopathy, miR-126 mimic-approach may represent a new therapeutic strategy in the prevention and treatment of DR and offer interesting prospects in future clinical application.

The main limitation of our findings is the absence of an animal model to extend the results obtained in vitro. However, the results of this study may contribute to develop clinical approach to counteract VEGF-A rise in response to hyperglycemia.

## Data Availability

The data used to support the findings of this study are included within the article.

## References

[1] Lee R., Wong T. Y., Sabanayagam C. (2015). Epidemiology of diabetic retinopathy, diabetic macular edema and related vision loss. *Eye Vision*.

[2] Wong T. Y., Cheung C. M., Larsen M., Sharma S., Simo R. (2016). Diabetic retinopathy. *Nature Reviews Disease Primers*.

[3] Zheng Y., He M., Congdon N. (2012). The worldwide epidemic of diabetic retinopathy. *Indian Journal of Ophthalmology*.

[4] Klein R., Klein B. E., Moss S. E., Cruickshanks K. J. (1994). Relationship of hyperglycemia to the long-term incidence and progression of diabetic retinopathy. *Archives of Internal Medicine*.

[5] Shin E. S., Sorenson C. M., Sheibani N. (2014). Diabetes and retinal vascular dysfunction. *J. Ophthalmic Vis. Res.*.

[6] Hammes H. P., Feng Y., Pfister F., Brownlee M. (2010). Diabetic retinopathy: targeting vasoregression. *Diabetes*.

[7] Apte R. S., Chen D. S., Ferrara N. (2019). VEGF in signaling and disease: beyond discovery and development. *Cell*.

[8] Behl T., Kotwani A. (2015). Exploring the various aspects of the pathological role of vascular endothelial growth factor (VEGF) in diabetic retinopathy. *Pharmacology Research*.

[9] Chang M. L., Chiu C. J., Shang F., Taylor A. (2014). High glucose activates ChREBP-mediated HIF-1*α* and VEGF expression in human RPE cells under normoxia. *Advances in Experimental Medicine and Biology*.

[10] Takahashi H., Shibuya M. (2005). The vascular endothelial growth factor (VEGF)/VEGF receptor system and its role under physiological and pathological conditions. *Clinical Science (London, England)*.

[11] Strauss O. (2005). The retinal pigment epithelium in visual function. *Physiological Reviews*.

[12] Kannan R., Zhang N., Sreekumar P. G. (2006). Stimulation of apical and basolateral VEGF-A and VEGF-C secretion by oxidative stress in polarized retinal pigment epithelial cells. *Molecular Vision*.

[13] Xiao Q., Zeng S., Ling S., Lv M. (2006). Up-regulation of HIF-1alpha and VEGF expression by elevated glucose concentration and hypoxia in cultured human retinal pigment epithelial cells. *Journal of Huazhong University of Science and Technology. Medical Sciences*.

[14] Ke Q., Costa M. (2006). Hypoxia-inducible factor-1 (HIF-1). *Molecular Pharmacology*.

[15] Bartel D. P. (2004). MicroRNAs: genomics, biogenesis, mechanism, and function. *Cell*.

[16] Fish J. E., Santoro M. M., Morton S. U. (2008). miR-126 regulates angiogenic signaling and vascular integrity. *Developmental Cell*.

[17] Liu B., Peng X. C., Zheng X. L., Wang J., Qin Y. W. (2009). MiR-126 restoration down-regulate VEGF and inhibit the growth of lung cancer cell lines in vitro and in vivo. *Lung Cancer*.

[18] Meng S., Cao J. T., Zhang B., Zhou Q., Shen C. X., Wang C. Q. (2012). Downregulation of microRNA-126 in endothelial progenitor cells from diabetes patients, impairs their functional properties, via target gene Spred-1. *Journal of Molecular and Cellular Cardiology*.

[19] Yang W. Z., Yang J., Xue L. P., Xiao L. B., Li Y. (2017). MiR-126 overexpression inhibits high glucose-induced migration and tube formation of rhesus macaque choroid-retinal endothelial cells by obstructing VEGFA and PIK3R2. *Journal of Diabetes and its Complications*.

[20] Livak K. J., Schmittgen T. D. (2001). Analysis of relative gene expression data using real-time quantitative PCR and the 2(-delta delta C(T)) method. *Methods*.

[21] Zhou Q., Anderson C., Hanus J. (2016). Strand and cell type-specific function of microRNA-126 in angiogenesis. *Molecular Therapy*.

[22] Zampetaki A., Kiechl S., Drozdov I. (2010). Plasma microRNA profiling reveals loss of endothelial miR-126 and other microRNAs in type 2 diabetes. *Circulation Research*.

[23] Ye P., Liu J., He F., Xu W., Yao K. (2014). Hypoxia-induced deregulation of miR-126 and its regulative effect on VEGF and MMP-9 expression. *International Journal of Medical Sciences*.

[24] Wang L., Lee A. Y. W., Wigg J. P., Peshavariya H., Liu P., Zhang H. (2016). miR-126 regulation of angiogenesis in age-related macular degeneration in CNV mouse model. *International Journal of Molecular Sciences*.

[25] Chen J. J., Zhou S. H. (2011). Mesenchymal stem cells overexpressing MiR-126 enhance ischemic angiogenesis via the AKT/ERK-related pathway. *Cardiology Journal*.

[26] Fang S., Ma X., Guo S., Lu J. (2017). MicroRNA-126 inhibits cell viability and invasion in a diabetic retinopathy model via targeting IRS-1. *Oncology Letters*.

[27] Pishavar E., Behravan J. (2017). miR-126 as a therapeutic agent for diabetes mellitus. *Current Pharmaceutical Design*.

